# Slower and Less Variable Binocular Rivalry Rates in Patients With Bipolar Disorder, OCD, Major Depression, and Schizophrenia

**DOI:** 10.3389/fnins.2019.00514

**Published:** 2019-05-21

**Authors:** Xing Ye, Ruo-Lin Zhu, Xiao-Qin Zhou, Sheng He, Kai Wang

**Affiliations:** ^1^Department of Neurology, The First Affiliated Hospital of Anhui Medical University, Hefei, China; ^2^Department of Neurology, Nanjing Brain Hospital, Nanjing Medical University, Nanjing, China; ^3^Fourth People’s Hospital of Hefei, Hefei, China; ^4^State Key Laboratory of Brain and Cognitive Science, Institute of Biophysics, Chinese Academy of Sciences, Beijing, China; ^5^Department of Psychology, University of Minnesota, Minneapolis, MN, United States

**Keywords:** binocular rivalry, perceptual dynamics, bistable perception, psychiatric disorders, endophenotype

## Abstract

When two different images are presented to the two eyes dichoptically, observers usually experience a perceptual alternation between the two images. This phenomenon, known as binocular rivalry, has been used as a powerful tool to investigate mechanisms of visual awareness. It was also found that the rates of perceptual alternation are slower in patients with bipolar disorder than in healthy controls (Pettigrew and Miller, 1998; Miller et al., 2003). To investigate the broader clinical relevance of binocular rivalry in psychiatric disorders, we measured the perceptual alternation rates during rivalry in healthy controls (*n* = 39) and in patients with different types of psychiatric disorders, including bipolar disorder type I (BD, *n* = 28), obsessive–compulsive disorder (OCD, *n* = 22), major depression (MD, *n* = 50), schizophrenia (SCZ, *n* = 44), and first-degree relatives (FDRs) of SCZ patients (*n* = 32). Participants viewed competing red–green images on a computer monitor through red–green anaglyph glasses and pressed buttons to record their perceptual alternations. The distributions of the rivalry rates were well described by a lognormal function in all groups. Critically, the median rate of perceptual alternation was 0.27 Hz for BD patients, 0.26 Hz for the OCD patients, 0.25 Hz for the MD patients, and 0.23 Hz and 0.27 Hz for the SCZ patients and their FDRs, respectively. All of which were significantly slower than the rate of 0.41 Hz obtained for the healthy controls, suggesting there may be shared genotypes between these different disorders. While rivalry alternations were generally slower in different types of psychiatric disorders compared to healthy controls, adding variance of rivalry rates in the analysis helped to partially separate among the different patient groups. Our results suggest that the slowing of binocular rivalry is likely due to certain common factors among the patient groups, but more subtle differences between different patient groups could be revealed when additional properties of rivalry dynamics are considered.

## Introduction

The visual brain consistently receives information based on retinal images from the two eyes and continuously integrates the two slightly different percepts into a unique interpretation of the visual world (Helmholtz and Southall, 1925). However, when two images presented to the corresponding retinal locations of the two eyes are incompatible, perception switches back and forth between the two different input images, a phenomenon known as binocular rivalry (Wheatstone, 1838; Levelt, 1965; Lehky, 1988; Blake and Logothetis, 2002). Binocular rivalry is a unique example of the more general phenomena of bistable perception, and in all cases a constant stimulus input leads to alternating perceptual interpretations. In bistable perception, since the stimulus remains unchanged, the spontaneous perceptual alternations reflect the intrinsic dynamic operations of the brain and potentially provide a tool to reveal the normal and abnormal dynamic properties of the functional brain.

Not surprisingly, visual stimulus features, such as luminance (Wolfe, 1983), spatial frequency (O’Shea et al., 1997), motion speed (Knapen et al., 2007), and stimulus size (Kang, 2009) can influence the temporal dynamic of binocular competition. However, when the physical features of the stimuli are not changed, a distinct property of bistable perception is that the rate at which perception switches is quite stable over time for an individual (George, 1936; Enoksson, 1963; Pettigrew and Miller, 1998; Miller et al., 2010), yet the switching rates are highly variable across individuals (Aafjes et al., 1966; Wang et al., 2014; Cao et al., 2018). A significant component of factors that determine the individual variation in perceptual switching rates is heritable, as demonstrated in twin (Miller et al., 2010; Shannon et al., 2011; Wang et al., 2014) and genomic (Chen et al., 2018) studies. In addition, brain imaging studies suggest that the dynamics in bistable perception are correlated with structure and function of focal brain areas such as the parietal and frontal cortex (Kleinschmidt et al., 1998; Lumer et al., 1998; Sterzer and Kleinschmidt, 2007; Kanai et al., 2010; Watanabe et al., 2014) and early visual areas (Yamashiro et al., 2014). Non-invasive brain stimulation using transcranial magnetic stimulation (TMS) also suggested that different subregions of parietal cortex may play different roles in influencing the rate of bistable perception (Carmel et al., 2010; Kanai et al., 2010, 2011). There is considerable evidence from pharmacological studies that show the dynamics of bistable perception are influenced by dopaminergic (Phillipson and Harris, 1984), GABAergic (van Loon et al., 2013) (but also see Sandberg et al., 2016) and serotonergic systems (Carter et al., 2005, 2007; Nagamine et al., 2008). Similarly, the dynamics of bistable perception can be modulated by central nervous system stimulants, with perceptual reversal rates increased by caffeine, but decreased by alcohol and sodium amytal (George, 1936).

Research on the dynamics of bistable perception has clear clinical relevance. Over the past several decades, many studies have consistently shown that subjects with pathological conditions have abnormal patterns of bistable perception compared with healthy subjects (Calvert et al., 1988; Pettigrew and Miller, 1998; Li et al., 2000; Miller et al., 2003; Krug et al., 2008; Nagamine et al., 2009; Robertson et al., 2013; Said et al., 2013; Freyberg et al., 2015; Xiao et al., 2018). One notable example is the slower rate of binocular rivalry alternations observed in patients with bipolar disorder (BD) (Pettigrew and Miller, 1998; Miller et al., 2003). Using both binocular rivalry and ambiguous structure-from-motion stimuli, the follow up studies strengthened the proposal that the reduced perceptual alternation rates could serve as an endophenotype of BD-I and bipolar spectrum disorder (Krug et al., 2008; Nagamine et al., 2009; Ngo et al., 2011; Vierck et al., 2013; Law et al., 2015). Moreover, studies have found slower switching dynamics for binocular rivalry in other clinical populations such as patients with schizophrenia (SCZ) (Sappenfield and Ripke, 1961; Fox, 1965; Frecska et al., 2003; Wright et al., 2003; Xiao et al., 2018), first-degree relatives (FDRs) of SCZ patients (Wright et al., 2003), and major depression patients (MD) (Meldman, 1965; Jia et al., 2015). In addition to psychiatric disorders, the slowing of bistable perceptual switching was also observed in migraine patients between migraine events (Wilkinson et al., 2008; McKendrick et al., 2011). However, slower binocular rivalry switching was not consistently observed in patient groups. For example, in a study that clearly demonstrated slower binocular rivalry switching in BD patients, SCZ and MD patients showed normal switching rates (Miller et al., 2003). In addition to binocular rivalry, other types of bistable stimuli have also been tested in clinical populations with mixed results. Slower perceptual switching of reversible figures was reported in children with corpus callosum pathology (Fagard et al., 2008). Viewing the Necker cube, subjects with generalized anxiety disorder (Meldman, 1965) and attention deficit hyperactivity disorder (ADHD) (Gorenstein et al., 1989) showed slower switching rate, but no such effect was observed in other studies (Li et al., 2000; Jusyte et al., 2018). When viewing bistable Schroeder’s figures patients with obsessive–compulsive disorder (OCD) experienced faster alternations than that of healthy controls, though the differences were not statistically significant (Li et al., 2000).

The inconsistency in experimental results described above may partially arise from issues such as diagnostic classification, disease comorbidity, or medication effects, but important factors to consider are differences in the experimental stimuli used and the behavioral recording protocols (Law et al., 2017). Therefore, a key aim of the current study is to use a consistent experimental stimulus and paradigm to examine the perceptual switching dynamics in patients with different types of psychiatric disorders for a better understanding of rivalry variability across different clinical populations.

First, we measured the perceptual alternation rates during rivalry in healthy controls and in patients with different types of psychiatric disorders (such as BD, OCD, MD, SCZ) and FDRs of SCZ patients. Additionally, we compared and selected lognormal function from three theoretical distribution functions to better fit the data of perceptual switching dynamics. In addition, we defined a two-dimensional parameter space to provide a more intuitive perspective to understand the effects and trends of different psychiatric disorders on switching rate dynamics. We also used statistical tools such as the Fisher discrimination analysis (FLDA) (Scholkopft and Mullert, 1999) and the standard bootstrapping procedure to highlight the differentiation of the different patient groups. Additionally, we investigated potential medication effect on binocular rivalry, albeit with a relatively small number of patients who were taking medications.

## Materials and Methods

### Observer

The subjects consisted of 39 healthy controls and 144 patients with different types of psychiatric disorders, including 28 subjects with BD type I (ICD-10, code F31), 22 subjects with OCD (ICD-10, code F42), 50 subjects with MD (ICD-10, code F32) and 44 subjects with SCZ (ICD-10, code F20). In addition, 32 FDRs of SCZ patients were also recruited for the study (Table 1). The BD patients were divided into two groups: 13 subjects with a main diagnosis of current episode mania (ICD-10, code F31.1) and 15 subjects with a main diagnosis of current episode depression (ICD-10, code F31.3). All patients were recruited from the outpatient clinics of the psychiatry department at the Fourth People’s Hospital in Hefei. All the patients were independently diagnosed by at least two deputy chief physicians from the department of psychiatry according to the ICD-10 criteria for research (World Health Organization [WHO], 1993). The patients had no history of neurological disorder, severe medical disorder, substance abuse, or electroconvulsive therapy. Patients with comorbid conditions were excluded from our study. The study included 32 FDRs (parents, siblings, and offspring) of SCZ patients. A specially designed set of screening criteria was adopted to ensure that all FDRs who met the inclusion criteria were recruited into the study. Inclusion criteria for FDRs were as follows: (1) Subjects of either sex; (2) age between 18 and 65 years; (3) no history of neurological illness; (4) at least primary school level of education and the ability to understand the requirements of the study. Exclusion criteria included: (1) lifetime history of any psychiatric disorder; (2) substance abuse; (3) history of head injury with cognitive sequelae or with loss of consciousness; (4) mental retardation; or (5) any medical illnesses that might significantly impair neurocognitive function. The 39 healthy controls (23–60 years of age, *y* = 39.74 ± 10.23; 21 females and 18 males) were recruited from among the university students and employees who were screened by a medical practitioner for symptoms of as well as personal and family history of psychiatric disorders. The controls were generally matched to the patient groups rather than specifically matched to each group (see Table 1 for details). The control group had slightly lower proportion of females than the patient group. However, randomly removing 4 male subjects from the control group (so that the proportion of female/male is well matched to the patient groups) produced no noticeable change in the results. The mean, SD, and range of the ages for each group are: BD (*n* = 28) 37.71 ± 9.06 (22–57); MD (*n* = 50): 39.24 ± 12.67 (15–64); OCD (*n* = 22): 35.37 ± 7.81 (25–52); SCZ (*n* = 44): 35.59 ± 11.33 (19–56); FDRs (*n* = 32): 47.03 ± 4.89 (41–58); Controls (*n* = 39): 39.74 ± 10.23 (23–60). Subjects had normal color vision (based on Ishihara color plate test), visual acuity (based on Snellen chart test, with acuity below 20/25 excluded), and did not have other visual impairments. Written informed consent was obtained from the participants of this study. All subjects were compensated for their participation. The study was approved by the Institutional Review Board of the Anhui Medical University. All subjects were naive to the purpose of the study. Each participant who underwent binocular rivalry rate measurement was free of caffeine and alcoholic beverages before testing (Shannon et al., 2011; Chen et al., 2018).

**Table 1:** Demographic information and binocular rivalry switching rates of different subject groups/subgroups.

Item	BD (*n* = 28)	BD subgroups		MD (*n* = 50)	OCD (*n* = 22)	SCZ (*n* = 44)	FDRs (*n* = 32)	Controls (*n* = 39)
			
		Manic mode (*n* = 13)	Depressive mode (*n* = 15)					
Age (years), mean ± SD	35.77 ± 9.06	34.54 ± 7.01	35.60 ± 10.53	39.24 ± 12.67	35.37 ± 7.81	35.59 ± 11.33	47.03 ± 4.89	39.74 ± 10.23
(range)	(22–57)	(23–46)	(22–57)	(15–64)	(25–52)	(19–56)	(41–58)	(23–60)
Female, n(%)	17 (60.71%)	8(61.54%)	9(60.00%)	38 (76.00%)	12 (54.55%)	26 (59.09%)	18 (56.25%)	21 (53.85%)
Catch Trial Accuracy, %	98.25 ± 4.09	98.52 ± 4.99	98.08 ± 3.13	98.59 ± 3.49	98.61 ± 4.01	97.34 ± 5.93	98.30 ± 6.02	99.38 ± 2.22
Trial1 vs. Trial3, r	0.7883^∗^	0.8117^∗^	0.7196^∗^	0.7414^∗^	0.9086^∗^	0.7754^∗^	0.7985^∗^	0.8552^∗^
Mean Rivalry Rate (Hz)	0.32 ± 0.02	0.30 ± 0.03	0.32 ± 0.03	0.29 ± 0.01	0.33 ± 0.04	0.31 ± 0.03	0.33 ± 0.03	0.44 ± 0.01
Median Rivalry Rate (Hz)	0.27 ± 0.01	0.26 ± 0.03	0.27 ± 0.03	0.25 ± 0.01	0.26 ± 0.03	0.23 ± 0.01	0.27 ± 0.02	0.41 ± 0.02
Mode (Hz)	0.22 ± 0.02	0.27 ± 0.03	0.18 ± 0.01	0.16 ± 0.01	0.17 ± 0.02	0.15 ± 0.01	0.18 ± 0.02	0.30 ± 0.01
Variance (Hz)	0.03 ± 0.00	0.03 ± 0.01	0.02 ± 0.00	0.05 ± 0.01	0.03 ± 0.01	0.10 ± 0.03	0.07 ± 0.02	0.07 ± 0.01


*All data are presented as the mean ± SEM. ^∗^*P* < 0.001.*

### Stimuli

**FIGURE 1:**
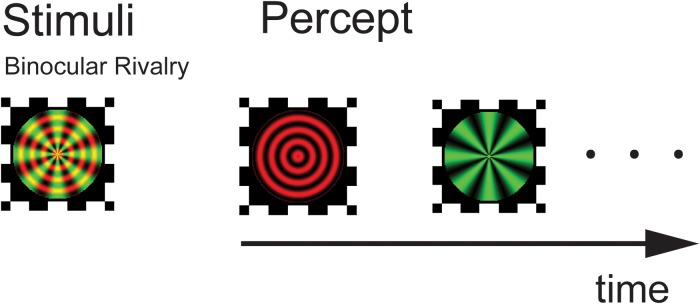
Experimental design and stimuli. Following a practice session, subjects viewed a composite of red circular and green radial gratings through a pair of red–green anaglyphic glasses. They perceived a stochastic alternation in percept between the two monocular images (binocular rivalry). Stimuli were presented for three 120-s trials with a 3 min rest period in between testing trials.

The experiment was conducted in a closed room with dim light. The visual stimuli were presented on a 14-inch monitor Lenovo laptop (1366 × 768 pixels; refreshment rate, 60 Hz; IdeapadZ580). Subjects were seated approximately 100 cm from the screen, yielding a viewing angle of 1.26° for the stimuli. The stimuli were composite images of a red concentric ring (8 cycles/deg) and a green radial grating (total 8 polar cycles in the pattern) with an average luminance of 135 cd/m2, sine wave modulated at a contrast level of 0.9. The colors on screen were adjusted in conjunction with the red–green filters so that there was minimal level of leaking across the two eyes. The stimuli were presented on dark gray background with luminance 30 cd/m2. A black frame (1.6° × 1.6°) that extended beyond the outer border of the stimulus was presented to facilitate stable fusion of the dichoptic stimuli (Figure 1). Relatively small stimuli and high contrast were used to help generate more clear-cut perceptual transitions. We used stationary stimuli in part to avoid the potential differential sensitivity to motion signals across different group of patients, as there is evidence of motion deficits in SCZ patients (Chen, 2011).

### Procedure

Participants viewed the superimposed red/green stimuli on the monitor through red–green anaglyph glasses (red filter over the right eye and green filter over the left eye). Responses were gathered with a standard keyboard. The stimulus presentations and trial timing were controlled using the MATLAB software package with Psychtoolbox (Lumer et al., 1998; Sterzer and Kleinschmidt, 2007).

Before starting the binocular rivalry test, a demo screen depicting the two alternating percepts was shown to the participant while the initial instructions were given. Participants were instructed to look at the center of the test images and press one button when the image of the green radial grating appeared and another button when the image of the red circular grating appeared. Subjects were instructed to press the appropriate button once their perception changed to predominantly the new percept (i.e., in the case of noticeable mixed percept, press the button when stimulus change passed the 50%). Following the practice session, participants viewed the dichoptic stimulus for three trials, with each trial lasting 120 s, separated by a 3 min break in which a blank screen appeared. To ensure the subjects’ compliance in the perceptual task, their performance was monitored by pseudo-randomly programmed catch trials in each test session. The rate of perceptual alternation was calculated as the number of rivalry switches divided by the total viewing time (seconds).

**FIGURE 2:**
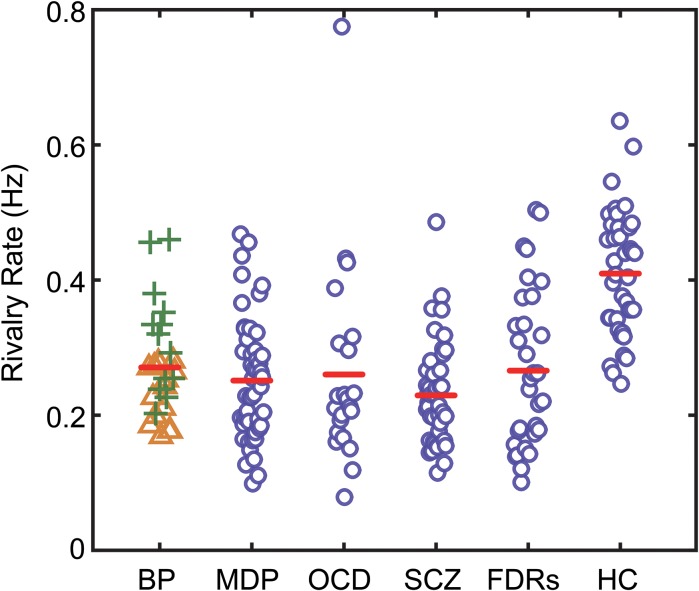
The binocular rivalry switching rates of all subjects in the present study. Switching rates for each subject are plotted according to their group. The red lines show the median values for each group. BD, bipolar disorder (green plus signs denote BD in manic phase, yellow triangle signs denote BD in depression phase); MD, major depression; OCD, obsessive–compulsive disorder; SCZ, patients with schizophrenia; FDRs, first-degree relatives of SCZ patients; HC, healthy controls.

**FIGURE 3:**
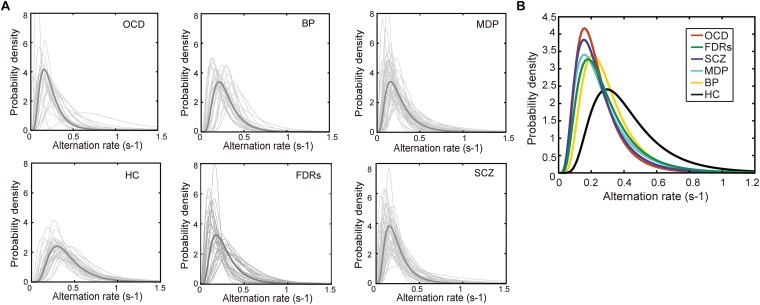
The probability density distribution of the binocular switching rates. **(A)** The probability density distribution of the alternation rates could be well described by a lognormal function in all 6 groups. Light gray line: the distribution alternation rates fitted by a lognormal function. Dark gray line: the lognormal distribution function generated from each group’s median parameters. **(B)** The lognormal distribution functions of the patient groups were significantly different from that of healthy control group. Each line stands for the median distribution of each group. Total numbers of fitted functions and subject numbers are BD: 1401, Subjects: 28; MD: 2514, Subjects: 50; OCD: 1022, Subjects: 22; FDRs: 1472, Subjects: 32; SCZ: 1707, Subjects: 44; HC: 2109, Subjects: 39.

In all subject groups, the subjects appeared to experience genuine binocular rivalry, as supported by three observations. First, the switching rates obtained from the different groups of subjects are in the range consistent with previous studies (see Figure 2). Second, the distribution of dominance times and switching rates conformed to the typical lognormal distribution (see Figure 3). Third, catch trials provided additional confirmation that participants were following instructions correctly. In each trial, we placed 3 periods of catch trials pseudo-randomly. During catch trials, the two monocular stimuli used during the binocular rivalry condition were alternately presented, each for between 0.5 and 5 s, to roughly simulate the perceptual transitions experienced during rivalry. If a participant pressed the correct button in response to the stimulus within an 800 ms time window, then that response was counted as a correct response. We calculate the proportion of their correct responses. Participants were instructed to press the button in response to the stimulus. All the subjects were provided with sufficient pre-experimental training to ensure that they were familiar with the task. They were asked to report their perceptual experience during the presentation of a 2 min movie of simulated rivalry to make sure that subjects understood the task requirements and can report their visual experiences accurately during all the experiment trials. The mean correct response rate for the catch trials was above 97%, confirming that the observers were following instructions.

## Results

Since age difference could potentially contribute to the observed difference in rivalry dynamics, we checked and found there was no correlation between the mean age and mean switching rates across the six groups of participants. To have more power for the correlational analysis, we pooled the data from the six groups and found that there was no correlation between the mean age and mean switching rates (*r* = 0.1719, *p* = 0.7448). We also checked the correlation between age and switching rates in each group and found that correlation coefficients ranged between 0.033 (BD) to 0.291 (FDRs), with none reaching significance level. At the group level, the data clearly showed that the patients, regardless of the specific type of psychiatric disorders they were suffering from, had slower binocular rivalry switching rates (Figure 2). Due to the variances not being homogeneous among groups (Levene’s test, *p* < 0.05), we used a one-way ANOVA (Welch’s *F* test for unequal variances) to test differences in the means of rivalry rates. There was a statistically significant difference between groups [*F*(5,84.83) = 19.821, *p* < 0.001].

Subsequently, Bonferroni-corrected *post hoc* tests of the rivalry rates between the healthy control subjects and the patients as well as the FDRs were performed. The results showed that all patient groups, as well as the FDRs group, had significantly slower rivalry rates compared to control group [*t*_MD_(87) = 7.569, *p* < 0.001; *t*_OCD_(59) = 4.861, *p* = 0.003; *t*_BD_(65) = 4.864, *p* < 0.001; *t*_SCZ_(81) = 9.751, *p* < 0.001; *t*_FDRs_(69) = 5.772, *p* < 0.001; respectively]. The switching rates of the two subgroups of BD patients were significantly different, with patients in the depressive mode having significantly slower rates than the patients in the manic mode [*t*_BD-subgroups_(26) = 3.344, *p* = 0.045, Bonferroni-corrected]. BD-depression group was significantly different from the healthy control group [*t*_BD-depression_(52) = 6.945, *p* < 0.001]. But BD-manic group was not significantly different from the healthy control group [*t*_BD-manic_(50) = 1.525, *p* = 0.727]. However, except for the two subgroups of BD patients, no statistically significant difference was found between all patient groups/subgroups. We also performed the Games-Howell test, which accommodates the unequal sample sizes and variances between samples. Results are consistent with the Bonferroni-corrected *post hoc* test.

It has been shown that the dynamics of binocular rivalry, especially the distribution of dominance durations or switching rates, could be well described by a gamma function (Levelt, 1965; Lehky, 1988; Kleinschmidt et al., 1998; Blake and Logothetis, 2002; Watanabe et al., 2014). There are other studies (Kanai et al., 2010; Yamashiro et al., 2014) which showed that lognormal and weibull distributions are actually better at describing perceptual alternations rates during binocular rivalry. We performed the goodness-of-fit tests (Kolmogorov–Smirnov one-sample test) to compare each of the experimental cumulative distribution functions to each of the three theoretical distributions (gamma, lognormal and weibull distributions). There were statistically significant differences between group mean p values as determined by one-way ANOVA [*F*(2,642) = 15.57, *p* < 0.001]. The result showed that the lognormal is better than gamma and weibull distributions [each group mean *p*-values of KS test: *p*(lognormal) = 0.5680, *p*(gamma) = 0.4580, and *p*(weibull) = 0.4016]. We then selected lognormal function from the three theoretical distribution functions to fit the data. Indeed, this was true in the current data as well, as shown by the conformation of the data distribution to a lognormal function both at the individual level and the group level (Figure 3). Two parameters were derived from the switching rate distribution of each subject: the mode, which indicated the most frequent value of his/her binocular rivalry switching rate, and the variance, which described the variability in his/her binocular rivalry switching rate. Specifically, fitting the switching rate data with a probability density function (formula 1) generated two parameter estimations, namely, μ (the location parameter) and σ (the scale parameter). Subsequently, the mode and variance parameters of a lognormal distribution could be obtained from the estimated μ and σ values (formulas 2, 3).

(1) $$f(x)=\frac{1}{\sqrt{2\pi \sigma x}}e^{-(\ln\;x-\mu )}{}^{{}^{2}/2\sigma^{2}}$$

(2) $$Mode(x)=e^{\mu -\sigma^{2}}$$

(3) $$Variance(x)=(e^{\sigma^{2}}-1)e^{2\mu +\sigma^{2}}$$

While the estimated mode values from the distributions and the calculated means of switch rates are highly correlated, using ‘mode’ in analysis provides a potential advantage in its resistance to extreme values in the distribution. One-way ANOVA was used to test differences in the mean parameters (mode and variance) of the lognormal distributions for all subject groups. We found significant differences between the groups for the mode parameter [mode: *F*(5,207) = 22.77, *p* < 0.001; variance: *F*(5,207) = 2.16, *p* = 0.602]. *Post hoc* comparisons using *t*-tests with Bonferroni correction indicated that the mean values of the mode parameter in the psychiatric disorder groups, as well as in the FDRs group, were significantly lower than that in the healthy control group. The estimated mode parameters for each group were as follows: 0.22 Hz in the BD group, *t*(65) = 4.229, *p* < 0.001; 0.16 Hz in the MD group, *t*(87) = 8.867, *p* < 0.001; 0.17 Hz in the OCD group, *t*(57) = 6.215, *p* < 0.001; 0.15 Hz in the SCZ group, *t*(81) = 9.331, *p* < 0.001; and 0.18 Hz in the FDRs group, *t*(69) = 6.625, *p* < 0.001.

**FIGURE 4:**
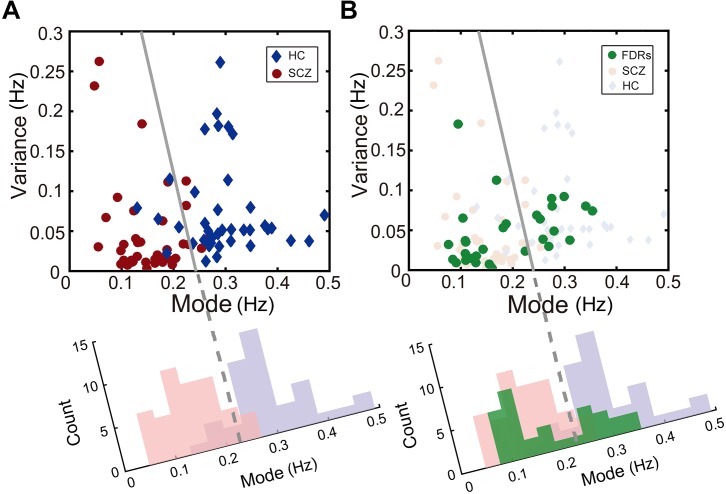
Description of binocular rivalry dynamics in the two-dimensional space defined by the mode and variance of rivalry switching rates. **(A)** SCZ patients (red dots) and healthy controls (blue dots) could be separated clearly in this two-dimensional space. **(B)** In addition, FDRs of patients with SCZ (green dots) apparently fall into two groups and are distributed on two sides of the boundary independently determined by Fisher’s linear discriminant (FLD) analysis on SCZ patients and healthy controls. The dots for each group were projected onto the axis orthogonal to the boundary line and the resulting univariate distributions show the count of points along the projected axis (SCZ in pink; healthy controls in lavender; FDRs in green).

Scatterplots are useful for revealing relationships between observations from different subject groups. For instance, the individual rivalry dynamics from the healthy control group and the SCZ group were plotted as scatterplots in two-dimensions (the mode and the variance), which turned out to be well segregated (left panel of Figure 4).

To quantify the accuracy of the binocular rivalry switching dynamics (characterized in terms of the mode and the variance of the switching rate distribution) used to discriminate between the healthy control group and each patient group, we applied Fisher’s linear discriminant analysis (FLDA) (Scholkopft and Mullert, 1999) method for multigroup classification. The quality of the linear boundary line produced by FLDA between the two groups can be quantified by the ratio (*w*-value) of the variance between the classes to the variance within the classes. Higher *w*-values indicate more robust classification (i.e., the boundary generated by FLDA produced better separation between the groups). Formula 4 defines the Fisher criterion, which is also known as the signal-to-interference ratio (SIR): *m* represents the projected mean of each group, and *s*^2^ represents the projected variance of each group. In the present study, after maximizing over all the linear projections, the classification performance between the SCZ group and the healthy control group showed a ratio of *w* = 2.45 [*t*(81) = 10.13, *p* < 0.001].

Interestingly, the data from the FDRs group appear to fall into two subgroups, separated by the linear discriminant boundary (Carmel et al., 2010) independently defined between the SCZ patients and healthy controls (right panel of Figure 4). When projected to the axis determined by maximizing the Fisher discriminant index, there is a suggestion of bimodal distribution for the FDRs group, but a larger sample size would be needed to support or refute this statistically.

Using a similar procedure, the discrimination between the other patient groups and the healthy control group showed a ratio of *w* = 2.05 [*t*(87) = 9.57, *p* < 0.001] for MD, a ratio of *w* = 2.61 [*t*(52) = 6.78, *p* < 0.001] for BD in the depression phase, a ratio of *w* = 0.49 [*t*(50) = 2.81, *p* = 0.0071] for BD in the manic phase, and a ratio of *w* = 1.56 [*t*(57) = 6.51, *p* < 0.001] for OCD.

(4) $$J(w)={\frac{\left|m_{2}-m_{1}\right|}{S_{1}^{2}+S_{2}^{2}}}^{2}$$

The *w*-values described above provide essentially the same measure of the classification performance as the more familiar discrimination sensitivity index *d*’ (*d*-prime, according to formula 5). Here μ and σ represent mean and standard deviation of each group respectively, *H* and *P* represent healthy control group and patient group. Similar to the *w*-value, the *d*’ provides an index of the separation between two distributions, but in units of standard deviation. We calculated *d*’ between the distribution of the healthy control group and the distribution of each patient group in two-dimensional space projected to an axis determined by maximizing the Fisher discriminant index (see Figure 4A for an example). In other words, higher *d*-prime scores represent greater sensitivity to distinguish between the two groups. The *d*’ of the classification for each patient group with the healthy group was 2.21 for the SCZ group, 2.29 for the BD in the depression phase group, 0.99 for the BD in the manic phase group, 1.77 for the OCD group, and 2.03 for the MD group.

(5) $$d^{\prime }=\frac{\mu_{H}-\mu_{P}}{\sqrt{\frac{1}{2}(\sigma_{H}^{2}+\sigma_{P}^{2})}}$$

**FIGURE 5:**
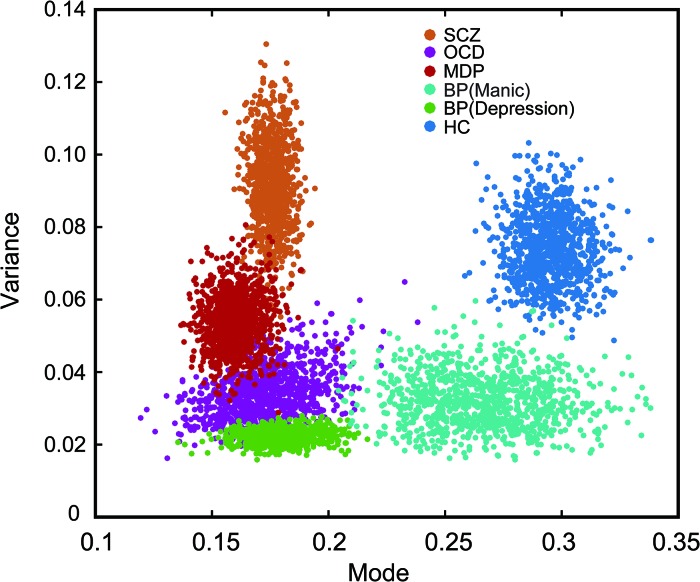
Bootstrapped plot of mode and variance from each group of subjects showing the central tendency of each group in the mode-variance space. Alternation rates were fitted separately for each participant with the best lognormal distribution. The two fitting parameters (mode and variance) from each group were then boot strapped (sampled 1000 times) to highlight the relationship between groups in this two dimensional plot. Note the individual dots are not denoting individual subjects, rather they are resampled data points. Different color markers were used for each group. The psychiatric disorder groups were significantly separated from the healthy control group.

To show the central tendencies of each group in the two-dimensional plot of the mode and the variance, we adopted a standard bootstrapping procedure (Phillipson and Harris, 1984; Kanai et al., 2011). The result demonstrated the distinct distribution of each participating group. To construct a bootstrap distribution for the mode and the variance, we first randomly selected individuals from each group with the same sample size (i.e., 10 participants in each group). Then the same procedure was repeated for *n* = 1,000 times with replacement (i.e., a participant could be selected more than once) to estimate the population means and variations for each group. The mean of this bootstrapped samples was then calculated and plotted as one of the points (x,y) in Figure 5, The horizontal (*x*) and vertical (*y*) axes showed the mode and the variance values of each group, respectively. As seen in Figure 5, data points of the healthy controls were well separated from that of the patient groups, however, data from some of the patient groups showed more overlap (e.g., between OCD and MD) (Figure 5).

**FIGURE 6:**
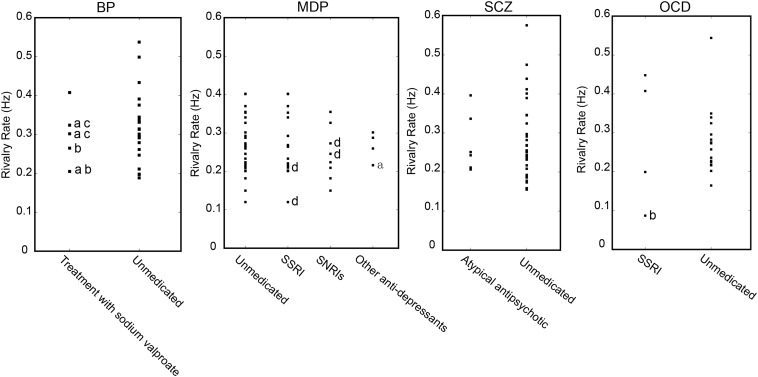
Potential effect of medication on the dynamics of binocular rivalry. Binocular rivalry switching rates are separately plotted for patients who received antipsychotic treatment and patients without any medication treatment. a, with SSRIs, selective serotonin reuptake inhibitor antidepressants; b, with an atypical antipsychotic; c, with an anxiolytic psychoactive drug; d, with NaSSAs, noradrenergic and specific serotonergic antidepressants.

A relatively small number of patients were under medication treatment, nonetheless we investigated the potential medication effect on binocular rivalry by comparing the switching rates between patients who received antipsychotic treatment and patients without any medication treatment (Figure 6). We also performed an additional ANOVA, which included all the unmedicated subgroups and the other medication treatment subgroups. No effect of medication on the alternation rate was observed [*F*(7,144) = 1.705, *p* = 0.093]. *Post hoc* comparisons using *t*-tests with Bonferroni correction indicated that difference between the two subgroups within each group was not significant in the rivalry rate for subgroup with medication treatment and subgroup without medication treatment. The comparison results for each group were as follows: in MD group, *t*(48) = 1.043, *p* = 0.961; in SCZ group, *t*(42) = 0.571, *p* = 0.571; in OCD group, *t*(20) = 1.445, *p* = 0.810; in BD group, *t*(26) = 0.266, *p* = 0.791.

## Discussion

Changes in the dynamics of bistable perceptual alternations have been previously reported in a number of studies of patients with psychiatric disorders, especially in BD patients during binocular rivalry (Pettigrew and Miller, 1998; Miller et al., 2003; Nagamine et al., 2007, 2009; Robertson et al., 2013; Vierck et al., 2013; Jia et al., 2015). In addition, there is still debate on whether a slow-down or speed-up in perceptual alternation rates would be observed in other psychotic patients, such as the slow-down in MD patients (Meldman, 1965; Jia et al., 2015) (but see Miller et al., 2003) and the slow-down in SCZ patients (Sappenfield and Ripke, 1961; Fox, 1965; Frecska et al., 2003; Wright et al., 2003; Xiao et al., 2018) (but see Miller et al., 2003). Different alternation rates between healthy controls and psychiatric disorder patients were also observed using other types of bistable stimuli, such as the Schroeder’s figures, the four-loop Lissajou figure and the Necker cube (Philip, 1953; Li et al., 2000; Hoffman et al., 2001). Investigation of the temporal dynamics of perceptual switching using a common stimulus and with the same parameters across a broad spectrum of psychiatric disorders, such as BD, MD, OCD, and SCZ, as well as the clinically unaffected FDRs of SCZ patients, provides a more comprehensive perspective and could potentially facilitate future optimal diagnoses and classification of psychiatric disorders.

Binocular rivalry is a widely used paradigm in the investigation of visual perceptual alternations. In the current study, we measured perceptual alternation rates during binocular rivalry in several types of psychiatric disorder patients and FDRs of SCZ patients. Our results showed that the slower perceptual switching rates than those of the healthy controls were not unique to BD but were also found in SCZ, MD, OCD and FDRs of SCZ patients. The alternation rates in our study ranged from 0.08 to 0.78 Hz, similar to those reported previously in the literature (Pettigrew and Miller, 1998; Miller et al., 2003, 2010).

Given that we used the same stimulus and also the same experimental paradigm across different patient and control groups, the finding that all patient groups showed slower binocular rivalry switching rate suggests there could be some common factors across the patient groups that contributed to the observed slowing of perceptual rivalry. The common factors could result from a high degree of genetic overlap between these psychiatric disorders (Lichtenstein et al., 2009; Cross-Disorder Group of the Psychiatric Genomics Consortium et al., 2013). On the difference between BD and SCZ (also MD and SCZ), our current results do not support their dissociation using rivalry switch rates, which is at odds with some previous findings (Miller et al., 2003). It is not clear what underlies the discrepancy between our findings and the conflicting previous findings. Both our study and that of Miller et al. (2003) used stationary, and thus relatively low-strength stimuli. It was suggested that higher-strength stimuli better distinguish BD from control subjects (Miller et al., 2003) so ultimately it would be appropriate for future specificity studies of binocular rivalry in different psychiatric disorders to also use higher-strength stimuli. There may have also been different characteristics of the SCZ and MD populations in the previous and current studies. Small sample sizes may also contribute to the conflicting findings and future studies will need to use larger sample sizes. Other protocol-related possible explanations for the conflicting findings are discussed further below.

In our study, patients with OCD also reported slower binocular rivalry switch rates. A previous study examined perceptual switching in OCD patients, but used bistable Schroeder’s figures rather than binocular rivalry (Li et al., 2000). They reported that subjects with OCD had a higher alternation rate than that of healthy controls though the differences were not statistically significant (Li et al., 2000). Because that study and the current study used different types of bistable stimuli, it is difficult to make a direct comparison, particularly in light of a recent study showing relatively low correlations in switch rates of different bistable stimuli and therefore possibly independent processing mechanisms underlying these different phenomena (Cao et al., 2018). Hence it is possible that binocular rivalry and ambiguous figures engage different cortical mechanisms and these mechanisms could be differentially affected by the presence of OCD.

We also examined switching rates according to the different mood states of BD (i.e., manic and depressive mood states). Our findings regarding BD in general are generally consistent with previous studies (Pettigrew and Miller, 1998; Miller et al., 2003, 2010; Nagamine et al., 2009; Vierck et al., 2013) in that the BD patients had slower switching rates than the healthy controls, irrespective of manic mood state. However, unlike some previous studies (Miller et al., 2003; Vierck et al., 2013), we found that depressive state did significantly modulate rivalry rate. Our result is similar to that of Jia et al. (2015) and Zhu et al. (2013), in showing that the depression state had a modulation effect on the rivalry rate. In addition, future research could further explore the differences in bistable switching rates under different clinical status and the correlation between rates and clinical status, with more comprehensive measures of bistable perception dynamics, including the periods of mixed or fused percepts. Follow-up study in a drug-naïve clinical population should be considered to explicitly discount medication confounding effects.

Through a further analysis of the perceptual switching dynamics, we could differentiate SCZ patients and other psychiatric disorders from healthy controls in two-dimensional space created by the mode and variance derived from the lognormal distribution of alternation rates. Interestingly, the rivalry dynamics of FDRs of SCZ patients had a wide spread. Referenced to the linear discriminant boundary line that best separated the SCZ group and the control group, the FDRs appear to fall into two subgroups, though a larger sample size will be needed to determine whether the FDRs distribution is in fact truly bimodal. In any case, these results support the idea that FDRs of SCZ patients may display cognitive symptoms characteristic of SCZ, and may be at a high genetic risk for developing SCZ compared with healthy controls (Salleh, 2004). Future genomic and longitudinal studies will be needed to investigate whether those FDRs of SCZ patients with slow rivalry rates share more susceptibility genes and are at higher risk of developing symptoms of SCZ.

Consistent with previous studies (Pettigrew and Miller, 1998; Miller et al., 2003; Nagamine et al., 2009), the slower switching rates in patients could not be explained by a medication effect. When all the medication-treated patients in all the psychiatric disorder type groups were excluded, there still existed the main effect of the slower switching rate on unmedicated patients. Indeed, the data from patients who were treated with medication were not distinguishable from those of patients with no medication intake (see Figure 6).

The dynamics of binocular rivalry is easy to measure, relatively stable across sessions in the same individual, and has a significant genetic contribution. Evidence coming from different studies has indicated that there is a great deal of symptomatic overlap and frequently shared susceptibility genes and pathways for psychiatric disorders (Craddock et al., 2006; Lichtenstein et al., 2009). With respect to the neural mechanism, given the observation that all the psychiatric disorder groups performed accurately in the catch trials and all groups had high between session correlations in switching rates, it is unlikely that their slow rivalry switches were caused by certain general cognitive factors, such as less attentive or alert. Previous studies have shown that psychiatric disorders are highly correlated with abnormalities in the genes coding for neurotransmitter receptors (such as glutamatergic and GABAergic receptors) or synaptic proteins (Blatt et al., 2001; Kegeles et al., 2012; Brady et al., 2013; van Loon et al., 2013; Cohen et al., 2015; Piantadosi et al., 2016; Chiu et al., 2017). For example, it was suggested that neurotransmitter imbalance and dysfunction – such as an excitation/inhibition (E/I) imbalance - may underlie general information processing, which could be generally more prominent in psychiatric disorder patients (Rubenstein and Merzenich, 2003; Canitano and Pallagrosi, 2017; Foss-Feig et al., 2017).

As noted above, there are several discrepancies between the current results and some of the previously published results, especially regarding whether binocular rivalry is also slower in SZ and MD patients in addition to the BD group (Miller et al., 2003; Vierck et al., 2013). There exist a number of differences between these studies in the stimuli and specific test protocols used and indeed the current study has certain protocol limitations that could be improved in future studies. For example, we did not measure the subjects’ perception of mixed percepts and were therefore not able to control for the confounding effect of mixed perception on binocular rivalry rate. Indeed, by incorporating mixed percepts into one or the other exclusive perceptual state, this would have a general effect of slowing of reported binocular rivalry rates. As such, it is possible that what appears in our data to be slowing of switch rate in the psychiatric disorders could in fact result from more mixed perception in some of these disorders. There is a precedent for more mixed perception in a clinical disorder, autism (Robertson et al., 2013). We had relatively short durations (6 min total) of rivalry measurements in our study, while some previous studies whose data conflict with ours used longer rivalry viewing periods (14 min total) and suggested that using stabilized rivalry rates is preferable (Miller et al., 2003).

The main strength of the current study was in its direct comparison across a broad spectrum of psychiatric disorders of switch rates with a common perceptual task. The differentiation between some of the disorders with the combination of the mean and variance of rivalry rates raises a feature of the dynamics of binocular rivalry that could be further explored in binocular rivalry endophenotype studies.

## Conclusion

We observed that the rivalry rates were slower in several groups of patients with psychiatric disorders than in a healthy control group, suggesting there may be shared genotypes between these different disorders. In the two-dimensional space formed by the mode and variance of switching rates, the different patient groups and the control group were more clearly segregated. Our results suggest that it will be helpful to include variance in the analysis of binocular rivalry dynamics in future large scale endophenotype research of psychiatric disorders.

## Ethics Statement

All subjects provided written informed consent and were compensated for their participation, as approved by the Institutional Review Board of the Anhui Medical University.

## Author Contributions

SH and KW designed the experiments. XY, R-LZ, and X-QZ performed the experiments. XY and SH analyzed the data and drafted the initial version of manuscript. XY and R-LZ finalized the manuscript with supervision from SH and KW.

## Conflict of Interest Statement

The authors declare that the research was conducted in the absence of any commercial or financial relationships that could be construed as a potential conflict of interest.
